# Assessing the Incidence of Ciguatera Fish Poisoning with Two Surveys Conducted in Culebra, Puerto Rico, during 2005 and 2006

**DOI:** 10.1289/ehp.1104003

**Published:** 2012-01-24

**Authors:** Eduardo Azziz-Baumgartner, George Luber, Laura Conklin, Thomas R. Tosteson, Hudson R. Granade, Robert W. Dickey, Lorraine C. Backer

**Affiliations:** 1 U.S. Centers for Disease Control and Prevention, Atlanta, Georgia, USA; 2 University of Puerto Rico, Mayagüez, Puerto Rico; 3 Food and Drug Administration Gulf Coast Seafood Laboratory, Dauphin Island, Alabama, USA

**Keywords:** ciguatera, ciguatera fish poisoning, ciguatoxins, incidence, poisoning, Puerto Rico, seafood

## Abstract

Background: Although ciguatera fish poisoning (CFP) is the most common seafood intoxication worldwide, its burden has been difficult to establish because there are no biomarkers to diagnose human exposure.

Objective: We explored the incidence of CFP, percentage of CFP case-patients with laboratory-confirmed ciguatoxic meal remnants, cost of CFP illness, and potential risk factors for CFP.

Methods: During 2005 and again during 2006, we conducted a census of all occupied households on the island of Culebra, Puerto Rico, where locally caught fish are a staple food. We defined CFP case-patients as persons with gastrointestinal symptoms (abdominal pain, vomiting, diarrhea, or nausea) and neurological symptoms (extremity paresthesia, arthralgia, myalgia, malaise, pruritus, headache, dizziness, metallic taste, visual disturbance, circumoral paresthesia, temperature reversal, or toothache) or systemic symptoms (e.g., bradycardia) within 72 hr of eating fish during the previous year. Participants were asked to save fish remnants eaten by case-patients for ciguatoxin analysis at the Food and Drug Administration laboratory in Dauphin Island, Alabama (USA).

Results: We surveyed 340 households during 2005 and 335 households during 2006. The estimated annual incidence of possible CFP was 4.0 per 1,000 person-years, and that of probable CFP was 7.5 per 1,000 person-years. One of three fish samples submitted by probable case-patients was positive for ciguatoxins. None of the case-patients required respiratory support. Households that typically consumed barracuda were more likely to report CFP (*p* = 0.02).

Conclusions: Our estimates, which are consistent with previous studies using similar case findings, contribute to the overall information available to support public health decision making about CFP prevention.

Ciguatera fish poisoning (CFP) is a toxin-induced illness associated with eating subtropical and tropical marine finfish that have accumulated ciguatoxins through their diet (Friedman et al. 2008). CFP is common around the globe between latitudes 35°N and 35°S (Lewis 2001), in places like Puerto Rico, the U.S. Virgin Islands, and the South Florida region (Holt et al. 1984; Lawrence et al. 1980; Lewis et al. 1981; Morris et al. 1982). There is evidence, however, that the range of ciguatoxic fish is expanding into the northern Gulf of Mexico and north along the U.S. Atlantic seaboard toward North Carolina [Centers for Disease Control and Prevention (CDC) 2009].

Ciguatoxins are produced by benthic dinoflagellates in the genus *Gambierdiscus* found on Caribbean and Pacific coral reefs (Bagnis et al. 1980). Herbivorous fish, feeding on the reef vegetation where *Gambierdiscus* is epiphytic, accumulate ciguatoxin precursors in their tissues. The toxins continue to bioaccumulate through the food web to large predatory reef fish, particularly barracudas, jacks, snappers, and groupers (Bagnis et al. 1980). Even with high levels of ciguatoxin, contaminated fish appear, taste, and smell normal. Ciguatoxins are not destroyed by cooking, freezing, or any form of preservation, and there is no reliable field test for ciguatoxins in fish tissue.

When humans eat ciguatoxic fish, they typically develop symptoms within 72 hr, often within 2–6 hr. Most persons with CFP experience gastrointestinal symptoms (e.g., abdominal discomfort, nausea, and diarrhea), which are generally accompanied by neurological symptoms (e.g., paresthesia, vision changes, temperature reversal, and perioral numbness) and sometimes cardiovascular symptoms (e.g., palpitations) (Holt et al 1984). One of the hallmarks of CFP is an apparent reversal of hot and cold sensations that may actually be induced by an enhanced sensitivity, reported as burning skin, to any extreme temperatures. Although CFP infrequently causes death (case fatality, 0.1–12%), neurological symptoms may persist for months and may evolve to a debilitating chronic condition (Friedman et al. 2008; Mines 1997; Palafox and Buenconsejo-Lum 2001).

CFP is typically self-limited, and victims often forgo treatment or treat themselves at home, particularly in areas where CFP is endemic. Treatment options are limited to supportive care; however, some studies indicate that intravenous mannitol administered within 72 hr of eating the ciguatoxic fish may help ameliorate symptom severity and may protect patients from chronic neurological effects (Friedman et al. 2008). To prevent CFP, health authorities in endemic communities may warn residents to avoid eating large reef fish or ban the sale of barracuda.

Determining the incidence and economic burden of CFP is critical in assessing its relative public health importance in the array of competing health priorities. Although CFP is the most common form of seafood intoxication worldwide, it has been difficult to estimate the associated disease and economic burdens. CFP is difficult to diagnose, cases are typically not reported to health authorities, and traditional surveillance systems identify only a small percentage of CFP case-patients with severe disease who seek medical care (Tester et al. 2010). In addition, there is no standard laboratory biomarker to confirm the presence of ciguatoxins in human clinical specimens. In this 2-year study of the island of Culebra, Puerto Rico, we explored the incidence of CFP, the percentage of CFP case-patients with laboratory-confirmed ciguatoxic meal remnants, the cost of CFP illness, and potential risk factors for CFP.

## Methods

*Study population.* The Municipality of Culebra is a small (about 12 square miles) island located off the northeastern coast of Puerto Rico. The island has 1,868 permanent residents (U.S. Census 2001) living in 1,024 housing units, plus tourists who visit on weekends and holidays. The island is accessible only by light aircraft or a 2-hr ferry ride, and because of its relative isolation, most residents seek primary health care at the island’s single government-subsidized clinic. The island of Culebra was selected as the study site primarily because it is a small, stable community from which we could derive baseline incidence of CFP and because it is typical of Puerto Rico’s coastal communities where locally caught fish are an important source of dietary protein.

*Door-to-door survey of Culebra households.* We conducted a survey of all households in Culebra during September 2005, a time of the year when local barracuda (*Sphyraena barracuda*) frequently test positive for ciguatoxins (Tosteson et al. 1988). Study teams conducted door-to-door surveys of every occupied household on the island, including single-family homes, apartments, and live-aboard boats. We excluded households whose members did not live in Culebra during the month before the interview because we believed they would not have been significantly exposed to local fish. Study teams made up to three attempts at different times of day to contact each household. In addition, to ensure that we contacted all permanent residents, we confirmed with neighbors that apparently vacant dwellings were actually unoccupied.

*Household- and individual-level data collection.* Upon identifying an adult within each of the island’s occupied households, we described the purpose of the study, obtained written informed consent, and administered a questionnaire in Spanish. The questionnaire included information about household demographics, whether household members ate locally caught fish, the amount and type of fish eaten, and any illnesses household members experienced within 72 hr of eating a fish meal during the previous year (September 2004 through August 2005). In addition to obtaining household-level information, we attempted to interview each person (or an available proxy) who became ill within 72 hr of eating fish to assess illness onset, symptoms, species, and actual size of the fish participants believed had made them sick, and associated treatment and medical care costs. We excluded children < 7 years of age from the individual-level survey because we believed they would be unable to provide reliable information.

If samples were available, we also requested samples of the fish meal that participants believed had made them sick within 72 hr of consumption. Participants were also asked to call study coordinators for ciguatera testing of fish meal remnants if they developed symptoms compatible with CFP ≤ 72 hr after eating fish during 2005–2006. We repeated the survey among all occupied households in Culebra during the next summer (June 2006) regardless of whether households had participated in the 2005 survey. During the second survey, we asked about illnesses that occurred since our last visit (i.e., within the prior 10 months).

*Laboratory analyses.* Fish samples eaten by all case-patients were frozen and shipped to the Gulf Coast Seafood Laboratory, U.S. Food and Drug Administration, and analyzed for ciguatoxins using methods described by Dickey and Plakas (2010) and Dickey (2008).

*Case definitions.* Using published information about CFP symptoms (Bagnis et al. 1979; Blythe 1994; Blythe et al. 2001), we identified two groups of case-patients. Cases with possible CFP reported three or more gastrointestinal symptoms (abdominal pain, vomiting, diarrhea, or nausea) plus at least one additional neurological symptom (extremity paresthesia, arthralgia, myalgia, malaise, pruritus, headache, dizziness, metallic taste, visual disturbance, circumoral paresthesia, temperature reversal, or toothache) or systemic symptom (e.g., bradycardia). Cases with probable CFP reported three or more gastrointestinal symptoms, three or more neurological symptoms, and at least one additional systemic symptom. Cases with laboratory-confirmed CFP were probable CFP case-patients who also had a positive test for ciguatoxins in a sample of fish meal remnant they believed had made them ill within 72 hr of consumption.

*Calculation of estimated CFP incidence.* We analyzed data as if the island’s population was an open cohort and aimed to determine whether the estimated incidence of CFP on Culebra was similar to estimates from previous studies (Hanno 1981; Morris et al. 1982). We estimated the annual incidence of probable and possible CFP by dividing the number of case-patients identified by the total person-time for each follow-up period. Because we followed participants during only 10 months, we multiplied the number of case-patients identified during 2006 by 0.83 (10/12) to estimate the number of person-years participants from the second survey contributed to the study.

*Exploring risk factors and economic burden.* We used Fisher’s exact test to explore households’ probabilities of having members with CFP based on their households’ fish consumption habits. We also explored the hypothesis that targeted methods of fishing (e.g., spear guns) allowed local fishermen to predominantly obtain fish they correctly believed, based on experience, to be nonciguatoxic because of their size or species. We estimated the direct and indirect costs of illness using standard World Health Organization (2010) CHOICE (CHOosing Interventions that are Cost Effective) methods. Investigators asked participants to recall direct costs associated with illness (physician or hospital visits, medications taken, and laboratory fees). To calculate a conservative estimate of indirect cost, we multiplied the minimum wage in Puerto Rico during 2004 (US$5.15; U.S. Department of Labor 2005) by the number of hours in a typical work shift (8 hr) and then by the number of days participants missed work because of their illness.

*Human subjects.* This project was reviewed and approved by the institutional review boards of Puerto Rico and the CDC. Investigators then obtained permission from the Puerto Rico Department of Health and the Municipality of Culebra before initiating the study. Culebra residents provided written informed consent in order to be eligible to participate in the study.

## Results

*Participating households.* During 2005, we identified 846 dwellings in Culebra, 497 (59%) of which were occupied. We spoke with adult representatives of all 497 households, and 340 (68%) agreed to participate in the study, 86 (17%) were unavailable after more than three visits, 41 (8%) were ineligible because household members did not typically eat fish, 15 (3%) were ineligible because household members did not live in Culebra the month before the interview, and 15 (3%) declined to participate. The 340 participating households comprised 893 household members (an average of 2.6 persons per household) 7–98 years of age (median, 40 years), 44% of whom were female (Table 1). During the 2006 follow-up, we enrolled 335 households with 1,007 household members 1–98 years of age (median, 34 years), 50% of whom were female.

**Table 1:** Demographics of participants in Culebra, Puerto Rico, surveyed during 2005–2006.

Characteristic	All participants (2005, *n* = 893; 2006, *n* = 1,007; 1,733 person-years)	Developed symptoms within 72 hr after eating fish (*n* = 39)	Possible case‑patient (*n* = 7)	Probable case‑patient (*n* = 13)
Age, years [median (interquartile range)]		38 (20–54)		49 (42–63)		51 (36–60)		46 (39–64)
Females [*n* (%)]*a*		876 (47)		17 (44)		3 (43)		6 (46)
Race [*n* (%)]								
White				32 (84)		5 (71)		11 (92)
African American		Not available		3 (8)		1 (14)		1 (8)
Other				2 (5)		1 (14)		
Refused				1 (3)				
Hispanic [*n* (%)]		Not available		37 (95)		6 (86)		13 (100)
Estimated annual incidence, per 1,000 person-years		Not applicable		10.8		4.0		7.5
**a**Of 1,901 with available sex information.								

*Household fish consumption.* Participating households typically ate between 0.25 and 3 lb (median, 1 lb) of fish per week (i.e., 6 oz of fish per household member per week). During 2005, 280 of 340 households (82%) ate locally caught fish, 32 (9%) ate imported fish, 25 (7%) ate both imported and locally caught fish, and 3 (1%) ate fish from unknown sources. During 2006, a similar percentage of households ate local fish (286/335; 85%). Of the 280 households that ate local fish during 2005, 114 (41%) typically caught the fish themselves, 72 (26%) received the fish as gifts from friends or family, 53 (19%) obtained fish from local fishermen, 26 (9%) purchased fish at local markets, 11 (4%) purchased fish at local restaurants, and 4 (1%) purchased fish at supermarkets. A higher percentage of households obtained fish from local fishermen (106/286; 37%) during 2006. Most of the 135 households who knew how their local fish were caught during 2005 ate fish captured with a fishing line (113; 84%), spear gun (19; 14%), trap (2; 1%), or net (1; 1%).

A greater percentage of households consumed snapper, grouper, hogfish, triggerfish, jack, trunkfish, and barracuda during 2006 than during 2005 (Table 2). Few participants (1%) ate sharks or skates during 2005 and 2006. The percentage of households that consumed fish weighing < 5 lb increased from 246 (72%) in 2005 to 266 (79%) in 2006. During 2005 and 2006, respectively, 166 (49%) and 182 (54%) households reported that they avoided eating barracuda because of its potential toxicity. In 2005 and 2006, respectively, 59 (17%) and 94 (28%) households reported that they avoided eating crevalle jack (*Caranx hippos*), and 5 (1%) and 7 (2%) avoided hogfish (*Lachnolaimus maximus*), because they believed that these fish might be ciguatoxic.

**Table 2:** Local fish eaten in Culebra, Puerto Rico, households surveyed during 2005 and 2006.

Households eating this species [*n* (%)]			Possible or probable CFP case-patients who ate this species before illness [*n* (% of 20 case-patients total)]*a*
Fish	2005 (*n* = 340)	2006 (*n* = 335)	
Barracuda		21 (6)		31 (9)		10 (50)
Hogfish		46 (14)		68 (20)		4 (20)
Cero		117 (33)		189 (56)		2 (10)
Snapper		69 (20)		86 (26)		1 (5)*b*
Grouper		69 (20)		76 (23)		1 (5)*b*
Jack		36 (11)		58 (17)		1 (5)
Triggerfish		33 (10)		59 (18)		
Trunkfish		28 (8)		54 (16)		
**a**Two case-patients ate unidentified fish. **b**The case-patient who ate snapper also ate grouper within 72 hr of developing CFP.						

*Estimated annual incidence of CFP.* During September 2004 through August 2005, 26 of 893 household members developed symptoms within 72 hr after eating fish, including 5 that were classified as case-patients with possible CFP (6/1,000 person-years) and 10 classified as having probable CFP (11/1,000 person-years). Similarly, during September 2005 through June 2006, 13 of 1,007 participants developed symptoms within 72 hr after eating fish, including 2 who were classified as case-patients with possible CFP (2 per 1,000 person-years) and 3 classified as having probable CFP (4/1,000 person-years). One of the three fish samples submitted by case-patients during 2006 was positive for ciguatoxins, one was negative for ciguatoxins, and one had insufficient material for testing.

*Demographics and exposure history of case-patients.* The mean age of the 20 possible and probable case-patients was 47 years, and 11 (55%) were male. Most ill persons were white (16; 80%) and Hispanic (19; 95%). Only 2 (10%) of the 20 were professional fishermen. Ten (50%) of the ill persons ate barracuda; 4 (20%), hogfish; 2 (10%), cero (*Scomberomorus regalis*); 1 (5%), crevalle jack; 1 (5%), yellow tail snapper and grouper; and 2 (10%), an unidentified fish. Seven (35%) of the 20 case-patients obtained the implicated fish from a friend or a family member, 4 (20%) caught it themselves, 4 (20%) bought it from a professional fisherman, 3 (15%) bought it at a restaurant, and 1 (5%) bought it at a market. Of the 9 implicated fish eaten by persons who knew how the fish was caught, 5 (56%) were caught with a line, and 4 (44%) with a spear gun at an average depth of 15 ft (range, 7–20 ft). Case-patients ate a mean portion size of approximately 143 cm^2^ and 3 cm thick.

*Clinical and laboratory findings of case-patients.* Case-patients developed gastrointestinal, neurological, and nonspecific symptoms, including nausea, vomiting, or abdominal pain (20; 100%), diarrhea (19; 95%), arthralgia (15; 75%), myalgia or headaches (15; 75%), numbness of the lips (12; 60%), paresthesias or dizziness (12; 60%), metallic taste (11; 55%), vision changes or sensory alterations of their hands (10; 50%), cramps (6; 30%), hot and cold temperature sensation disturbances (5; 25%), or palpitations (5; 25%). None of the case-patients required respiratory support, and no fatalities occurred during the CFP events.

*Health seeking and associated direct and indirect costs.* Of the 20 possible and probable case-patients, 13 (65%) sought care within 72 hr of symptom onset, 2 (10%) sought care after 72 hr, and 5 (25%) did not seek care. Eight (40%) of the 20 case-patients received intravenous fluid, 6 (30%) received other unspecified treatment, 3 (15%) received no treatment, 2 (10%) provided no treatment information, and 1 (5%) received intravenous mannitol. Of the 15 case-patients who sought care, 11 (73%) attended the public clinic in Culebra, 2 (13%) did not specify where they sought care, 1 (7%) attended a private clinic in Puerto Rico, and 1 (7%) visited a local folk healer. Two (13%) were later referred to specialty care by the clinical team that first evaluated them. Ten (67%) of the 15 case-patients who sought care were diagnosed with “intoxication,” 2 (13%) with dehydration, 2 (13%) with other unspecified diagnoses, and 1 (7%) with CFP. The median direct cost of seeking health care was $0 (range US$0–28). Case-patients missed work for a median of 4 days (range, 0–14 days) during their illness, resulting in a median indirect cost of US$164.80 per episode (based on a minimum hourly wage of US$5.15).

Many of the case-patients reported that they took action to avoid exposure to ciguatoxic fish after becoming ill. Although 3 (15%) of the 20 case-patients did not change their fish-eating habits after their illness, 11 (55%) avoided eating fish that were known to be associated with CFP, 2 (10%) avoided eating fish from waters where ciguatoxic fish had been caught in the past, 2 (10%) avoided fish completely, and 2 (10%) changed their habits in other nonspecified ways.

**Figure 1:**
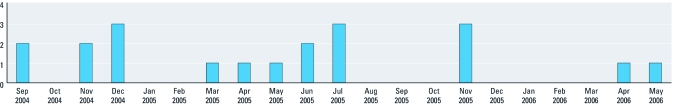
Timing of possible and probable CFP case-patients in Culebra, Puerto Rico.

*Household risk factors for CFP.* Most (19 of 20; 95%) possible and probable case-patients were able to identify the species of fish they ate just before becoming ill. During 2005 and 2006, 12 of 169 households (7%) that typically ate barracuda reported that a household member had experienced CFP, compared with 16 of 503 households (3%) that did not consume barracuda (*p* = 0.02). We found no association between fish size, method of capture, or source of fish and subsequent CFP. We examined the month that people reported becoming ill with possible or probable CFP (Figure 1), but we did not identify a seasonal trend.

## Discussion

We estimate that approximately 2–11 in 1,000 persons in Culebra, Puerto Rico, develop at least some symptoms associated with CFP each year (i.e., possible and probable CFP case-patients). The rate of CFP in Culebra is comparable to those of other common food-borne illnesses, such as nontyphoid salmonella infections (Voetsch et al. 2004). In addition, our estimate was similar to estimates derived from emergency department records in the U.S. Virgin Islands by Morris et al. (1982) and Hanno (1981) (7.3 cases per 1,000 person-years and 12 cases per 1,000 person-years, respectively) but higher than those reported by Escalona de Motta et al. (1986) and Lawrence et al. (1980). Specifically, Escalona de Motta et al. (1986) used emergency department records in Puerto Rico to estimate an incidence rate of 8–11 CFP cases per 10,000 person years, assuming that identified case-patients represented 10–15% of the total CFP cases in the community. Lawrence et al (1980) also assumed that passively reported CFP among mostly health-care–seeking case-patients in Miami Dade County represented one-tenth of case-patients in the community when deriving their estimated incidence rate of 5 cases per 10,000 person-years. Both of these studies may have underestimated the true incidence of CFP by overestimating the percentage of health-care seeking among CFP cases.

In our study, most case-patients classified as having possible or probable CFP sought care at a government-subsidized clinic, and all seemed to have self-limited CFP. Case-patients had similar symptom complexes, severity, and management as described in other studies (e.g., Friedman et al. 2008; Holt et al 1984). Although none of the case-patients in our case-series developed severe adverse effects, most developed symptoms sufficiently severe to compel them to seek care. Similar to reports in the literature, approximately 40% received intravenous fluids (Holt et al. 1984), and only one case-patient was given mannitol (McKee et al. 2001), the treatment of choice for acute CFP (Friedman et al. 2008). Most case-patients did not incur direct costs from their illness because they were able to attend a government-subsidized clinic in Culebra. CFP events on average were associated with 4 days of productivity loss and a concomitant indirect cost. In our study, respondents indicated that they typically avoided eating barracuda, hogfish, or jack because of potential toxicity. As in other studies (Lawrence et al. 1980), however, respondents who did report eating barracuda and jack were more likely to report household members with symptoms compatible with CFP than those who did not report eating these species.

This study has important limitations. Mild CFP cases that occurred during the study period may not have been identified. We were only able to test three fish samples provided by case-patients. The development of assays to identify ciguatoxin biomarkers in human urine, blood, or other clinical sample would be useful for CFP case identification. We were unable to identify a spring and summer seasonality as was suggested by a review of case-patients identified through poison control centers (Holt et al. 1984). Our cost estimates do not include costs to the provider, an important source of economic burden in a setting where there is government-subsidized health care.

## Conclusions and Recommendations

Our study confirms previous findings indicating that CFP is a frequent occurrence in coastal communities such as Culebra, Puerto Rico, where reef fish are a food staple. Although all case-patients had self-limited disease, the severity of symptoms was sufficient to compel patients to seek free medical care. Only one case-patient was diagnosed with CFP, which suggests that even providers in endemic areas need continuing medical education about the diagnosis and management of CFP.

The decay of marine ecosystems and limited treatment options for CFP underscore the importance of continued active monitoring and implementation of preventive measures (Goater et al. 2011). CFP may be preventable if people avoid eating large reef fish, particularly barracuda. The government of Puerto Rico could consider enforcing the ban on the sale of barracuda, amberjack, and blackjack that was instituted in Puerto Rico during 1981. In addition, a public health campaign to urge island residents and tourists to avoid eating frequently ciguatoxic local reef fish may help reduce the incidence of CFP.
